# Improved Hardness and Thermal Stability of Nanocrystalline Nickel Electrodeposited with the Addition of Cysteine

**DOI:** 10.3390/nano10112254

**Published:** 2020-11-13

**Authors:** Tamás Kolonits, Zsolt Czigány, László Péter, Imre Bakonyi, Jenő Gubicza

**Affiliations:** 1 Department of Materials Physics, ELTE Eötvös Loránd University, Pázmány Péter sétány 1/A, H-1117 Budapest, Hungary; jeno.gubicza@ttk.elte.hu; 2 Centre for Energy Research, Institute for Technical Physics and Materials Science, Konkoly-Thege M. út 29-33, H-1121 Budapest, Hungary; czigany.zsolt@energia.hu; 3 Wigner Research Centre for Physics, Konkoly-Thege út 29-33, H-1121 Budapest, Hungary; peter.laszlo@wigner.hu (L.P.); bakonyi.imre@wigner.hu (I.B.)

**Keywords:** electrodeposition, nickel, cysteine, microstructure, hardness, thermal stability

## Abstract

Experiments were conducted for the study of the effect of cysteine addition on the microstructure of nanocrystalline Ni films electrodeposited from a nickel sulfate-based bath. Furthermore, the thermal stability of the nanostructure of Ni layers processed with cysteine addition was also investigated. It was found that with increasing cysteine content in the bath, the grain size decreased, while the dislocation density and the twin fault probability increased. Simultaneously, the hardness increased due to cysteine addition through various effects. Saturation in the microstructure and hardness was achieved at cysteine contents of 0.3–0.4 g/L. Moreover, the texture changed from (220) to (200) with increasing the concentration of cysteine. The hardness of the Ni films processed with the addition of 0.4 g/L cysteine (∼6800 MPa) was higher than the values obtained for other additives in the literature (<6000 MPa). This hardness was further enhanced to ∼8400 MPa when the Ni film was heated up to 500 K. It was revealed that the hardness remained as high as 6000 MPa even after heating up to 750 K, while for other additives, the hardness decreased below 3000 MPa at the same temperature.

## 1. Introduction

Nanocrystalline materials such as electrodeposited nickel [1,2,3,4] are frequently investigated due to their unique properties as compared to their coarse-grained counterparts [5,6]. Both the correlation between the physical properties and the microstructure of electrodeposited nickel and the effect of deposition parameters (such as pH and temperature of the bath, applied current density, stirring rate, and composition of the bath) were intensively studied during the past decades. A short summary of these studies can be found in our previous article [7].

Reduction of the grain size and increase of the defect density in nanocrystalline materials usually improve the hardness [8], but decrease the thermal stability [9], especially in the case of pure metals [9,10,11,12,13]. If alloying with other elements [14,15,16,17,18,19] or codeposition of secondary-phase particles (e.g., SiC) [20,21,22,23] are not applicable; the only way to hinder the grain growth during annealing is the application of additives during electrodeposition, which causes the inclusion of a small amount of impurities in the deposited layer. Even a small impurity content can improve the thermal stability [9,24,25,26,27], due to the segregation of impurity atoms to the grain boundaries and decreasing both the boundary mobility [28,29,30,31] and the extra enthalpy of the material caused by the presence of grain boundaries [12,32].

Cysteine is considered to be a promising additive to create nanocrystalline nickel with a very small grain size and with an improved thermal stability, due to the following reasons:

- As demonstrated earlier, incorporating sulfur can reduce the grain size, and increase the hardness [7,8,33]. Sulfur may also yield a moderate strengthening during annealing at low temperatures (between 400 K and 600 K) [9,24]. Cysteine contains sulfur and can easily decompose into sulfur-containing components [34]. Since the sulfur atom in the cysteine molecule is relatively weakly bound as compared to the most common additive saccharin, cysteine can be a good candidate as sulfur-donor bath additive.
- Additives with –SH functional groups (i.e., thiols) tend to adsorb on metal surfaces with the sulfur atom as the anchoring entity of the adsorbed molecule. Thiols can even form monolayers that block the direct interaction between the metal and the solution. This phenomenon has a rich literature [35,36,37,38,39], especially for noble metals like Au and Ag. Although this “organic shield” does not completely block the electron transfer between the metal and the reactive solute species, the adsorbed layer inhibits the growth of the already existing grains of the deposit and promotes the formation of new nucleation sites [7]. It has been well evidenced that the metal deposition can take place through the defect sites of adsorbed thiol layers [35,36,37,38,39]. Additionally, the interaction of the polar/ionic groups of the cysteine molecule (i.e., the amino and the carboxylate groups) can stabilize the solution side of the adsorbed layer by pinning the adsorbed molecules with zwitterion formation.
- Cysteine can easily form complexes with Ni [40,41,42].

The electrodeposition of different metals, such as Cu [43], CuZn [44] or As [41], in the presence of cysteine has already been studied. Investigations were conducted on the electrodeposition of gold with cysteine addition. Different morphologies like monocrystaline surfaces [45], nanoparticles [46,47] and dendrites [48] were observed. In the case of Ni deposited from cysteine containing baths, mainly the electrokinetic behaviour was investigated [42,49]. Although, Ebadi et al. concluded that cysteine could reduce the grain size and the surface roughness of Ni [49], a detailed analysis of the microstructure and its thermal stability is missing from the literature.

The aim of the present study was to investigate the effect of various cysteine concentrations in a nickel-sulfate based bath on the microstructure and hardness of Ni electrodeposits. At one selected cysteine concentration (0.4 g/L), the evolution of the defect structure (dislocation density and twin fault probability), grain size and hardness of Ni films were investigated at different annealing temperatures up to 1000 K. The microstructure was studied by the complementary usage of transmission electron microscopy (TEM), electron backscatter diffraction (EBSD) and X-ray line profile analysis (XLPA).

## 2. Materials and Methods

### 2.1. Electrodeposition of Nickel Samples

In order to investigate the effect of cysteine on the microstructure of Ni electrodeposit, first cysteine was added to an organic additive-free nickel electrolyte denoted as “NOA” and described in our former paper Ref. [8]. Then, it turned out that the original pH and current density conditions were unfavourable when cysteine was added to bath “NOA”, since the as-deposited samples were fragmented and had a poor adhesion to the substrate. Therefore, several preliminary trials were performed to find more appropriate electrodeposition conditions. On the basis of these experiments, a direct current density of $j=-25\;{\mathrm{mA}/\mathrm{cm}}^{2}$ and an increased concentration of boric acid were selected and the pH was adjusted to 6.1 using NaOH. Crack-free Ni films with a thickness as large as ∼30 µm could be successfully deposited by adjusting the deposition time, assuming 96% current efficiency [50]. The composition of the new bath is given in Table 1.

Samples were deposited at room temperature onto an 8 µm thick rolled Cu sheet acting as the cathode at the bottom of a $8\times 20\;{\mathrm{mm}}^{2}$ tubular cell [51]. Nickel resupply was provided by a soluble Ni anode. After deposition, the substrate was removed with electrochemical dissolution in the same way as described in Ref. [9]. The samples were denoted as CYS00, CYS01, CYS02, CYS03 and CYS04, corresponding to the cysteine contents of 0.0, 0.1, 0.2, 0.3 and 0.4 g/L, respectively. In this study, the notations “es” and “ss” were used to distinguish between the electrolyte and substrate sides of the as-deposited films.

### 2.2. Heat Treatment

As sample CYS04 (produced with a cysteine content of 0.4 g/L) exhibited the highest hardness (see section “Results” below), this material was used for the study of the effect of cysteine on the thermal stability of the as-deposited Ni nanostructure. Therefore, CYS04 samples were heated up to the temperatures of 400, 500, 600, 750 and 1000 K in an Ar gas atmosphere using a Perkin-Elmer 2 type differential scanning calorimeter (DSC) (Waltham, MA, USA). These temperatures were the same as the values used for the investigation of the thermal stability of Ni films processed with other bath additives in our former study [9], since we wanted to compare the influence of cysteine on the stability with the effects of other additives. The samples were heated up to the selected temperatures at a rate of 40 K/min, and then were cooled down to room temperature at a rate of 300 K/min (this is the highest applicable rate in the calorimeter used). Since there was no significant difference between the two sides “es” and “ss” of sample CYS04, after the heat treatment, only the electrolyte side (“es”) was studied. It should be noted that, in the heat treatment experiments, the parts of the same film were used for annealing.

### 2.3. X-ray Diffraction

X-ray diffraction (XRD) characterization of the microstructure consisted of three kinds of measurements: (i) phase analysis, (ii) X-ray diffraction line profile analysis (XLPA) of the lattice defect structure and (iii) study of the crystallographic texture by pole figures.

The phase analysis was performed on the XRD patterns taken by a Smartlab X-ray diffractometer (Rigaku, Tokyo, Japan), using CuK_α_ radiation (wavelength λ=0.15418nm). These measurements were taken in Bragg–Brentano geometry. The texture of the samples was characterized by the same Smartlab diffractometer, applying parallel-beam optics and in-plane geometry (referred to as pole figure measurements). The XLPA measurements for the characterization of the defect structure were performed by a RA-MultiMax9 rotating anode diffractometer (Rigaku, Tokyo, Japan) (with CuK_α1_ radiation, wavelength λ=0.15406 nm). The diffraction patterns were evaluated by the extended convolutional multiple whole profile (eCMWP) fitting method [52,53]. A brief description of this method is given below. During this evaluation, the whole experimental diffraction pattern was fitted by the sum of a background and theoretical peak profile functions. The latter profiles were obtained as the convolution of the measured instrumental peak and the calculated XRD profiles caused by the finite crystallite size, dislocations and twin faults. The instrumental profiles were measured on a LaB_6_ standard material. The peak profile function related to the crystallite size effect was calculated assuming a log-normal distribution of spherical-shaped crystallites [7], which was characterized by the median (m) and the log-normal variance ($\sigma^{2}$). The peak profile related to the strain effect depended on the density of dislocations (ρ) and their arrangement, while the function related to twin faults was influenced by the twin fault probability. The latter quantity is defined as the fraction of twin boundaries among the (111) lattice planes, denoted as β. From the fitting of the XRD patterns, the mean crystallite size, the dislocation density and the twin fault probability were determined. Details of the eCMWP procedure can be found in Refs. [52,53].

It is important to note that the size of the coherently scattering domains provided by the XLPA evaluation (consequently called “crystallite size” in this paper) is often smaller than the size of the regions bounded by high-angle grain boundaries (HAGBs) determined by transmission electron microscopy (TEM). The latter quantity is consequently called “grain size” in this paper. The difference between the grain and crystallite sizes is pronounced for Ni films with larger grain sizes and is manifested in an increasing *d*/<*x*> ratio with grain size, as depicted in Figure 10 of [8].

### 2.4. Transmission Electron Microscopy

TEM samples were prepared with mechanical polishing and Ar^+^ ion-beam milling with the use of a Technoorg Linda ionmill (Technoorg Linda, Budapest, Hungary). Thinning was started at high energy (10 keV) and was finished at an ion energy of 3 keV in order to minimize the structural changes induced by the ion beam [54]. A detailed description of sample preparation can be found in Ref. [7].

To investigate both sides of the as-deposited samples with different cysteine contents, cross-sectional (cv) samples were prepared. Since there was no difference between the two sides of the annealed samples, for this films, only the electrolyte side was investigated by plane view (pv) samples. The TEM measurements were performed by a Philips CM20 transmission electron microscope (Thermo Fisher, Waltham, MA, USA) operated at an acceleration voltage of 200 kV. Selected area electron diffraction (SAED) patterns were taken at a camera length of 1 m on areas with linear dimensions between 1 and 5 µm.

### 2.5. Scanning Electron Microscopy and Energy-Dispersive X-ray Spectroscopy

The chemical composition of the samples was investigated by energy-dispersive X-ray spectroscopy (EDS) using a Hitachi TM4000Plus scanning electron microscope (SEM) (Tokyo, Japan) equipped with an AztecOne EDS system (made by the Oxford Instruments – Bognor Regis, UK). The grain size for the sample heated up to 1000 K was studied by electron-backscatter diffraction (EBSD) using a FEI Quanta 3D SEM (Hillsboro, OR, USA). The size of the grains was determined from the grain maps obtained by EBSD using the linear intercept method.

### 2.6. Hardness Tests

Vickers hardness tests were performed by a Zwick/Roell ZHµ indenter (Ulm, Germany), using a load of 10 g. The investigated samples were not polished since this surface treatment could modify the as-deposited and the annealed microstructures. In order to avoid the influence of the substrate on the hardness values, the load was selected to a sufficiently small value so that it yielded indentation diagonal not higher than 66% of the thickness of the film (according to ISO 6507 standard). Thus, the indentation diagonal for all samples was kept to be less than 15 µm, since the film thickness was about 30 µm. Fifty indentations were made on each sample.

## 3. Results

### 3.1. The Effect of Cysteine Addition on the Chemical Composition and the Microstructure

Within the detection limit of the EDS analysis (∼0.1 at.% for both metallic impurities and sulfur), no foreign element was found in the samples deposited in the absence of cysteine, i.e., these specimens were pure nickel. In contrast, all the Ni samples deposited from baths with cysteine contained about 0.6–0.7 at.% sulfur irrespectively of the cysteine concentration of the bath.

**Figure 1:**
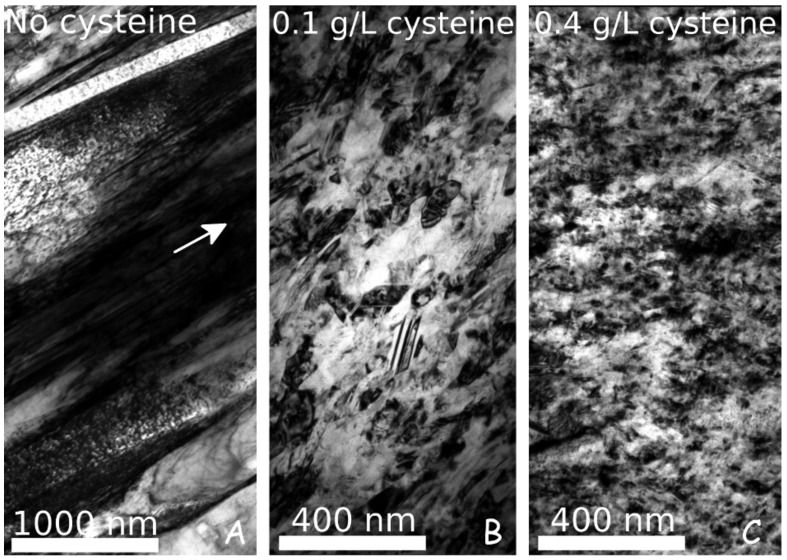
TEM images showing the microstructures in the cross section of the Ni films (close to the electrolyte side) processed without cysteine (**A**), 0.1 g/L cysteine (**B**) and 0.4 g/L cysteine (**C**). The white arrow in (**A**) indicates the growth direction of the film.

**Figure 2:**
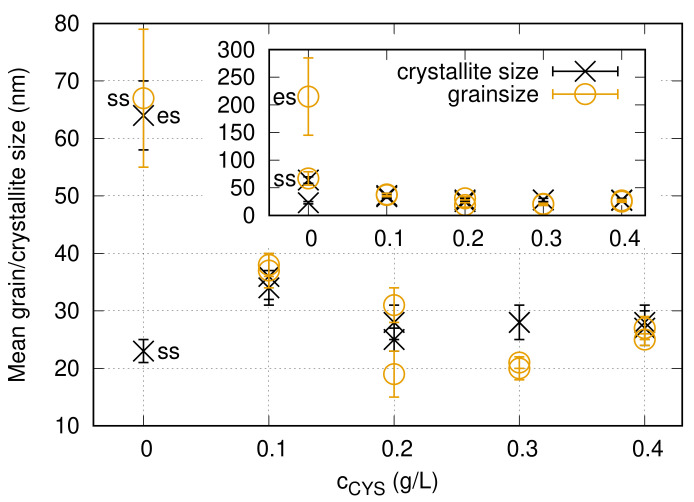
Grain size determined by TEM and crystallite size obtained by XLPA for the “es” and “ss” sides of the films versus the cysteine content of the bath. Subfigure was inserted to reveal the details of the figure without the outlier data point corresponding to the grain size of the electrolyte side of sample CYS00.

Figure 1 illustrates the effect of cysteine on the microstructure of Ni electrodeposits. These TEM images were taken on the cross-section of the films close to the electrolyte side. Although the TEM study was performed for all cysteine concentrations, Figure 1 shows only the cysteine-free sample and the films processed with 0.1 and 0.4 g/L cysteine, illustrating the trend of grain refinement with increasing additive concentration. The grain size values obtained from the TEM images (dTEM) for both the “es” and “ss” sides are listed in Table 2 and plotted in Figure 2. For the cysteine-free sample (CYS00), a columnar grain structure developed with increasing column diameter toward the “es” side. The column width (called grain size hereafter) was about 67 nm at the “ss” side, which increased to about 215 nm at the “es” side. With the addition of 0.1 g/L cysteine to the electrolyte, the grain size decreased to about 37 nm and the difference between the electrolyte and substrate sides of the sample disappeared. Columnar microstructure was not observed in the TEM images taken on the films deposited from the baths containing cysteine. With an increasing amount of cysteine in the bath, only a slight decrease in the grain size was observed and for the highest cysteine concentration applied (0.4 g/L), the grain size decreased to 25–27 nm.

The crystallite sizes obtained by XLPA for the different cysteine contents are listed in Table 2. For the cysteine-free case, the crystallite size values for both sides of the film are smaller than the grain sizes determined by TEM with a factor of about three. A similar effect has been observed formerly for materials in which the grains were divided into subgrains (see Ref. [8]). As XRD is sensitive to small misorientations, the crystallite size characterizes rather the size of subgrains and not the grains. For the films processed from the bath containing cysteine, the values of the crystallite size are similar to the grain size (see Table 2), which suggests that the grains with the size of about 20–30 nm are not divided into subgrains.

It is noted that the TEM investigations confirmed the log-normal shape of the size distribution function assumed in the XLPA evaluation. Namely, Pearson’s chi-square test [55] revealed that the grain size distributions for the different samples follow log-normal distribution, with a probability higher than 95% in most of the cases. A comparison between the directly determined median of the data and the calculated median assuming a log-normal distribution was also made, and the difference between them was always smaller than 5%. A detailed description of this evaluation procedure can be found in Ref. [8].

The dislocation density and the twin fault probability determined by XLPA are summarized in Table 2. For both sides of the cysteine-free sample, the dislocation density was about $34-39\times 10^{14}\;m^{-2}$, while the twin fault probability was zero. The latter observation is not in contradiction with the appearance of twins in the cross-sectional TEM image (see the top of Figure 1A). These twin boundaries are commonly perpendicular to the film surface (i.e., parallel to the diffraction vector), therefore they are practically invisible to the XLPA method. In addition, their average spacing is higher than the detection limit of XLPA (about 200 nm). It should also be noted that the microstructure of this cysteine-free layer differs significantly from the values obtained for the additive-free film used in our former studies (denoted as “NOA” in Ref. [8]). Table 3 shows the difference between the conditions of electrodeposition and the microstructural parameters for these two additive-free samples. The dislocation density for sample CYS00 is much higher than that for the film NOA that might have contributed to the higher hardness of the former film.

**Figure 3:**
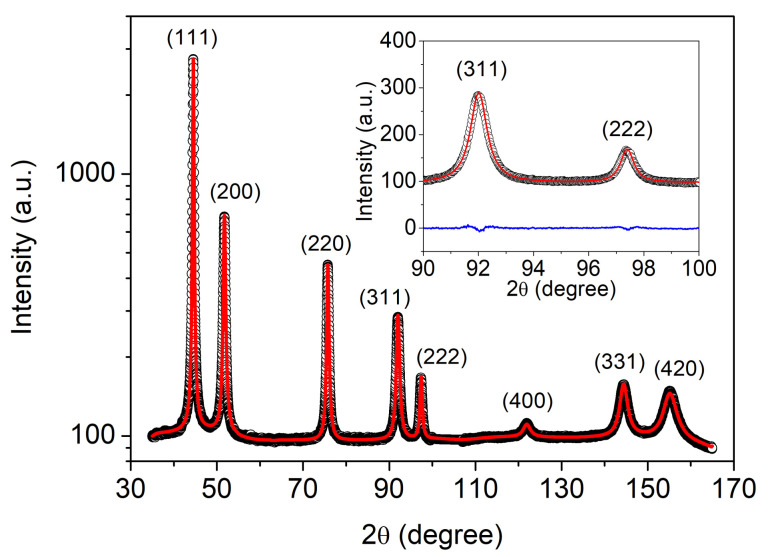
eCMWP fitting on the pattern taken on the “ss” side of the film CYS01. The black open circles and the red solid line represent the measured and the calculated XRD patterns, respectively. The intensity is displayed on a logarithmic scale. The inset shows a magnified part of the pattern with linear intensity scale. The difference between the measured and the calculated data is represented by the blue line.

**Figure 4:**
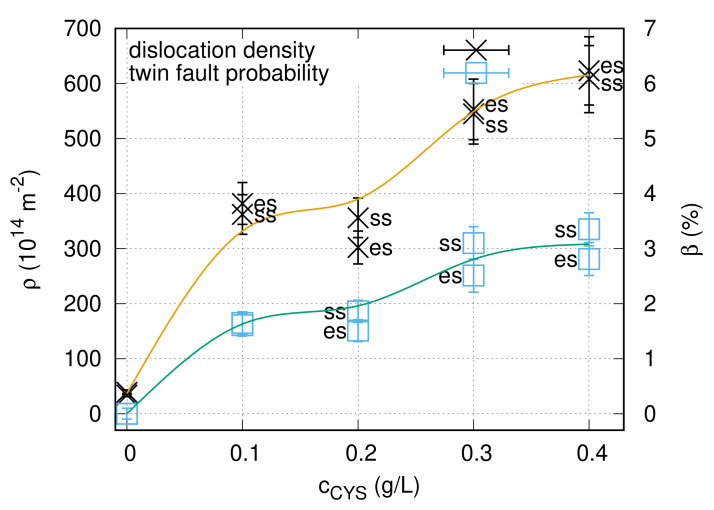
Dislocation density (ρ) and twin fault probability (β) versus the cysteine content of the bath for the “es” and “ss” sides of the films. The line serves as a guide for the eye only.

The density of lattice defects (dislocations and twin faults) was determined by XLPA using the eCMWP fitting method. Figure 3 illustrates the eCMWP fitting for the substrate side of sample CYS01. The evolution of the dislocation density and the twin fault probability obtained by XLPA versus the cysteine content in the bath is plotted in Figure 4. The addition of 0.1 g/L cysteine to the bath resulted in one order of magnitude enhancement of the dislocation density from ∼39 $\times 10^{14}\;m^{-2}$ to $360-380\times 10^{14}\;m^{-2}$. In addition, the twin fault probability increased from 0 to 1.6±0.2%. Both the dislocation density and the twin fault probability increased monotonously with the addition of cysteine. Initially, the increase was intense and then the saturation of both parameters was observed for the higher cysteine concentrations. The saturation value was about $620\times 10^{14}\;m^{-2}$ for the dislocation density and about 3% for the twin fault probability.

**Figure 5:**
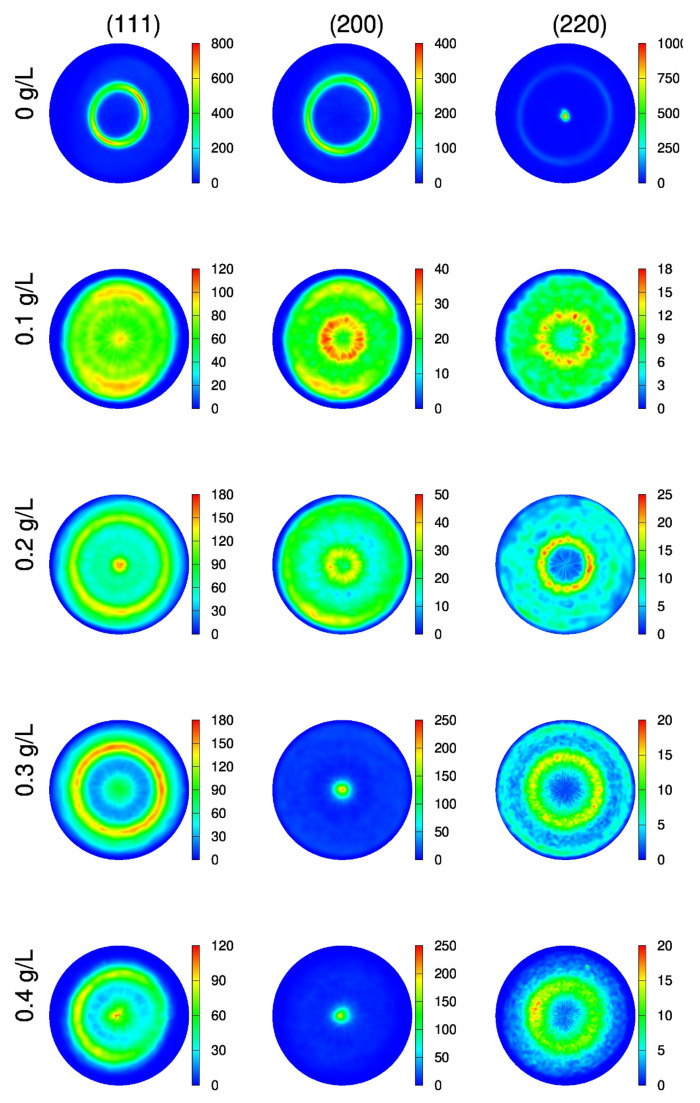
(111), (200) and (220) pole figures obtained by XRD for the “es” side of the Ni films deposited with different cysteine contents.

Figure 5 shows the evolution of the crystallographic texture on the “es” side of the films with increasing the cysteine content of the bath. Without cysteine, a (220) out-of-plane fiber texture was formed. When 0.1–0.2 g/L cysteine was added to the bath, a (111) texture was developed. With increasing the concentration of cysteine to 0.3–0.4 g/L, the dominating texture turned into a (200) one with a weaker (111) component. On the “ss” side of the Ni films, a similar texture evolution was observed with the increase of cysteine content.

### 3.2. Hardness Evolution with Increasing Cysteine Content

**Figure 6:**
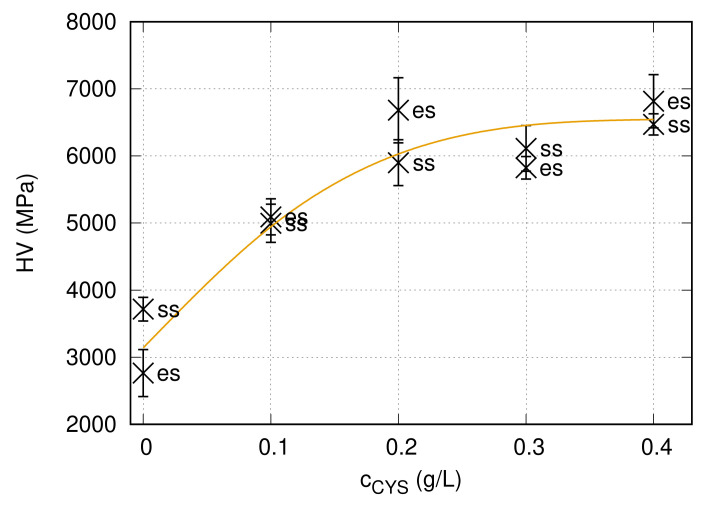
The Vickers hardness (HV) as a function of the cysteine content in the bath for the “es” and “ss” sides of the Ni films. The line serves as a guide for the eye only.

The hardness values obtained on the “es” and “ss” sides of the Ni films processed from the baths containing different amounts of cysteine are listed in Table 2. For the cysteine-free sample, the hardness on the side “ss” was higher (∼3700 MPa) than that measured on the “es” side (∼2800 MPa), most probably due to the smaller grain size. For the films deposited from the baths containing cysteine, a difference between the hardness values obtained on the two sides was not observed. The change of the hardness as a function of the cysteine content is shown in Figure 6. The hardness initially increased monotonously with the addition of cysteine. Initially, the increase was intense. Saturation of the hardness was observed for the higher cysteine concentrations, with a saturation value of about 6800 MPa.

### 3.3. Thermal Stability of the Microstructure in the Ni Film Processed with the Cysteine Content of 0.4 g/L

**Figure 7:**
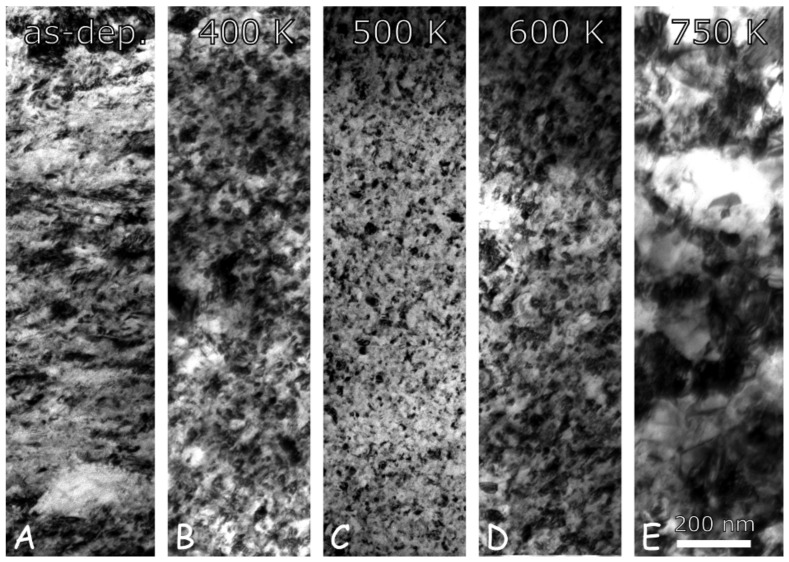
Plane view TEM images showing the microstructures on the “es” side of the CYS04 film in the as-deposited state (**A**) and after annealing at 400 (**B**), 500 (**C**), 600 (**D**) and 750 K (**E**).

**Figure 8:**
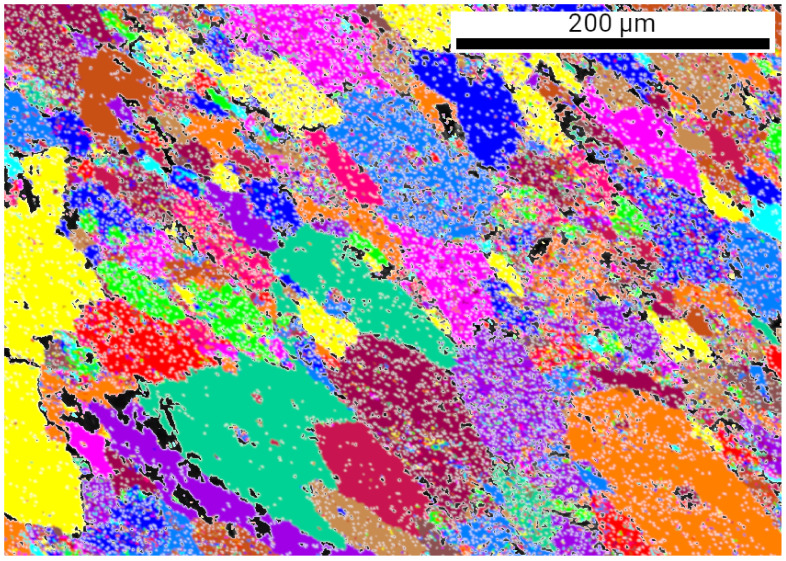
EBSD image on the microstructure obtained on the “es” side of the CYS04 film heated up to 1000 K.

**Figure 9:**
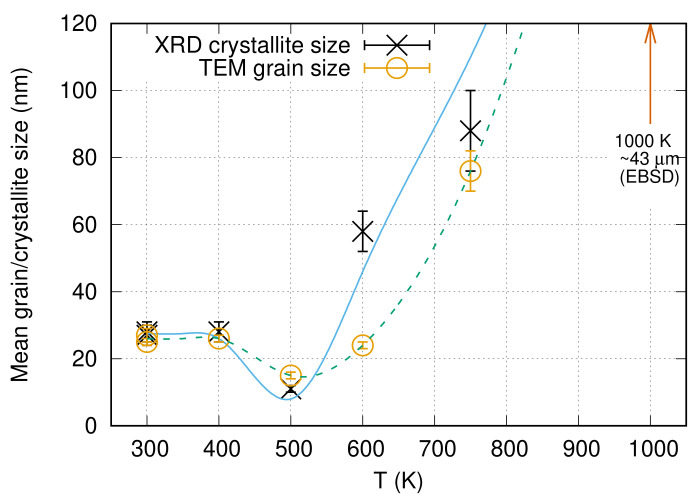
Grain size determined by TEM and crystallite size obtained by XLPA for the “es” side of the film CYS04 versus the annealing temperature. The line serves as a guide for the eye only.

**Figure 10:**
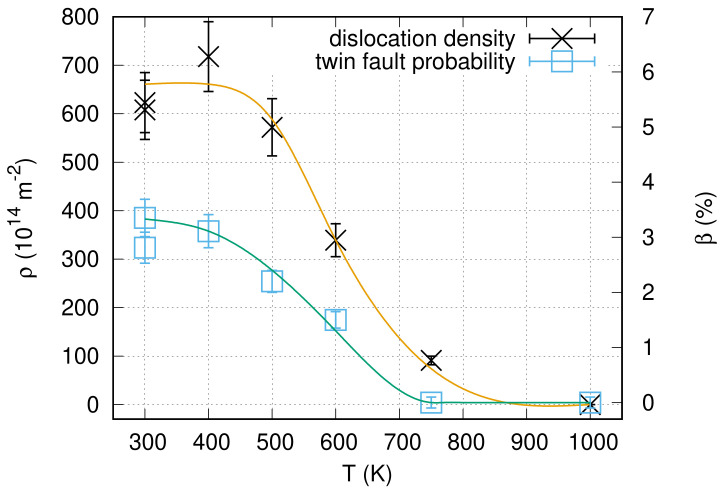
Dislocation density (ρ) and twin fault probability (β) for the “es” side of the film CYS04 versus the annealing temperature. The line serves as a guide for the eye only.

Figure 7 shows TEM micrographs taken on the “es” side of CYS04 sample heated up to 400, 500, 600 and 750 K. The microstructure obtained on the film heated up to 1000 K is depicted by the EBSD image in Figure 8. The grain sizes determined from the TEM and EBSD images are listed in Table 4 and plotted as a function of temperature in Figure 9. It can be seen that the grain size remained unchanged after heating up to 400 K. This is also true for the dislocation density and the twin fault probability (see Figure 10). When the annealing temperature was increased to 500 K, a slight decrease in the defect densities was observed. In addition, both the grain and crystallite sizes determined by TEM and XLPA decreased to about half of their original values. A further increase of the heat treatment temperature resulted in a gradual enhancement of the grain and crystallite sizes, as well as a simultaneous reduction of the dislocation density and the twin fault probability. It is worth noting that for 600 and 750 K, the crystallite size determined by XLPA is slightly different from the grain size determined by TEM. This observation can be explained by the fact that in the present case, the TEM characterized the grain size parallel to the film surface while XLPA determined the crystallite size perpendicular to the layer due to the applied diffraction geometry. Thus, the difference between the crystallite and grain sizes may be caused by the formation of grains elongated along the thickness after annealing at 600 and 750 K. Between 750 and 1000 K, the sample was fully recrystallized, which was indicated by the grain growth to 43 µm and the decrease of the defect density under the detection limit of XLPA ($10^{13}\;m^{-2}$ and 0.1% for the dislocation density and the twin fault probability, respectively).

**Figure 11:**
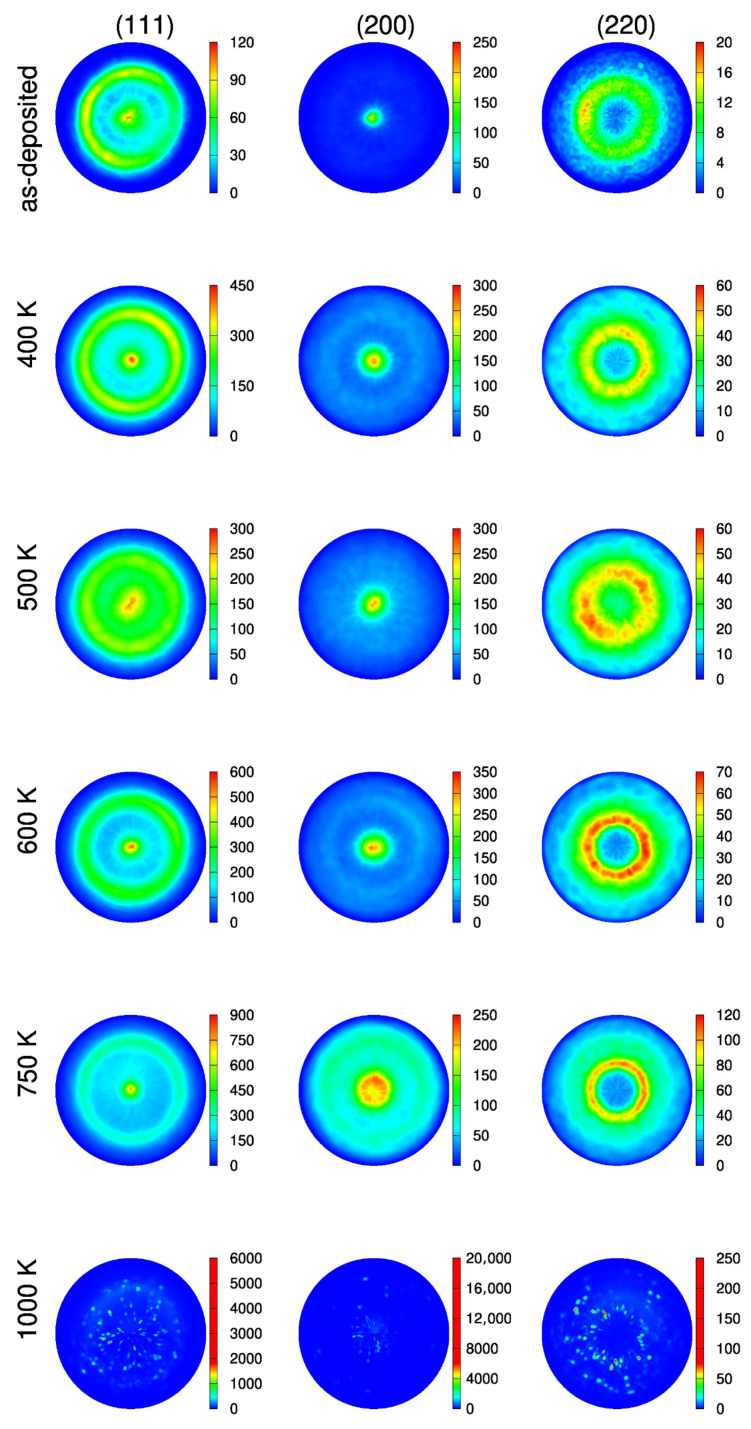
(111), (200) and (220) pole figures obtained by XRD for the “es” side of the CYS04 film heated up to different temperatures.

During the heat treatment of sample CYS04, both texture components (200) and (111) remained strong and component (111) took the dominance (see Figure 11). It should be noted that after heating up to 500 K, both texture components are less sharp than for either 400 or 600 K.

### 3.4. Change of the Hardness During Annealing of the Ni Deposited With Cysteine Additive

**Figure 12:**
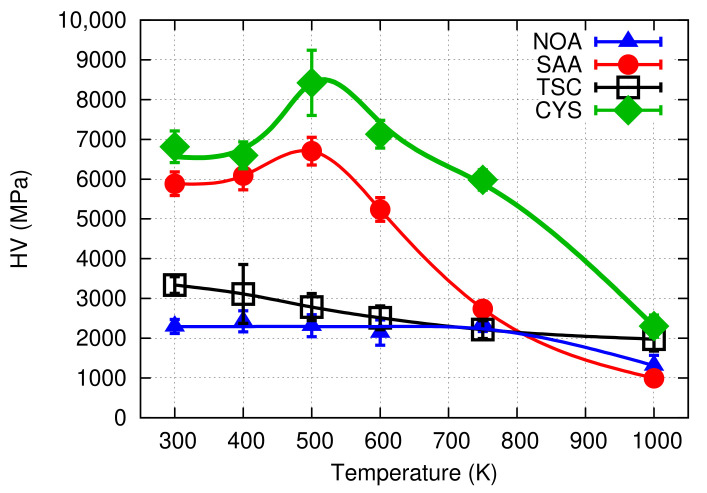
Vickers hardness (HV) as a function of the annealing temperature for the “es” side of the film CYS04 (denoted as CYS). In addition, the hardness evolution versus the temperature is also plotted for other additives, published formerly in Ref. [9]. NOA: no additive, SAA: saccharin, TSC: trisodium-citrate. The line serves as a guide for the eye only.

Figure 12 shows the evolution of the hardness as a function of the temperature of heat treatment. The hardness values are also listed in Table 4. Up to 400 K, a significant change in the hardness was not observed. At the same time, after heating up to 500 K, the hardness increased from ∼6800 MPa to ∼8400 MPa. At higher temperatures, the hardness gradually decreased, but even at 750 K, the hardness still had a high value of about 6000 MPa. For the highest temperature of 1000 K, the hardness decreased to ∼2300 MPa (see Table 4). The sample annealed to 500 K exhibited the most interesting behaviour, therefore this experiment was repeated on another film. The same microstructural parameters and hardness values were obtained in both cases.

## 4. Discussion

### 4.1. Effect of Cysteine Addition on the Texture, Grain Size and Defect Density

The grain size of an electrodeposited metal is determined by the ratio of the rates of new grain nucleation and growth of the existing grains. During electrodeposition of nickel in the presence of cysteine, there are various ways to reduce nickel: (i) reduction of a free (hydrated) Ni^2+^ ion, (ii) reduction of a Ni^2+^ ion formerly part of a Ni-cysteine complex [40,49]. The first process is certainly dominant, since the concentration ratio of Ni^2+^:cysteine is larger than 300. However, the surface is covered by the adsorbed cysteine molecules, independently of the Ni deposition process. A similar behaviour was demonstrated for Au-thiol adsorbate systems [45,47,56]. Here, we have to count with Ni-thiol surface groups, which is analogous to the Au-thiol adsorbates [57,58,59,60,61]. The surface coverage with the thiols means that the near-equilibrium growth sites are mostly blocked by the adsorbed molecules, and the maintenance of the growth rate with the constant current must lead to the nucleation of new crystallites that are not covered with an adsorbate layer at the moment of their formation, but also become thiol-covered soon thereafter.

During the deposition of a face-centered cubic (fcc) material such as Ni, the different crystal planes grow at different rates due to the surface energy anisotropy. The order of formation rate is (111) > (100) > (110) [62]. However, since nickel electrodeposition is always accompanied by hydrogen codeposition (because of the value of the equilibrium potential of the Ni(II)/Ni redox system), these formation rates can change [8,62,63]. In the absence of other strongly adsorbing additives, the different lattice planes have different hydrogen adsorption affinity, resulting in different hydrogen coverages [63,64,65]. Hydrogen codeposition favours the crystal planes with lower planar packing density. Thus, during electrodeposition of additive-free nickel, due to the hydrogen codeposition, the order of the formation rate of the crystalline planes is (110) > (100) > (111), which is in accordance with the experimentally observed strong (220) texture in specimen CYS00.

As mentioned above, during the electrodeposition of Au in the presence of cysteine, cysteine molecules could covalently attach to the metal surface in a preference order (110) > (100) > (111), causing an (111) type texture, since cysteine blocks the growth of the strongly covered crystallographic planes. Assuming the same effect for Ni, the strong (111) texture formed in the presence of a small amount of cysteine (0.1–0.2 g/L) can be understood.

When increasing the amount of cysteine in the bath, other planes (e.g., the less favoured (111) planes) are also covered by cysteine molecules, thereby blocking the growth of these planes, too. The effectiveness of this blocking depends on the number of the attached molecules (which favours the order 110 > 100 > 111) and the packing density of lattice points on the planes (which increases in the reverse order, 111 > 100 > 110). Thus, the combination of these two effects can result in the dominant (200) texture observed for higher cysteine concentrations (0.3–0.4 g/L).

Moreover, quantum mechanical calculations proved that during co-adsorption of S and H on Ni deposits, the adsorption of S at a three-fold site on plane (111) has a tendency to block the adsorption of H at the nearby surface sites [66], thus suppressing the effect of hydrogen codeposition on the (111) planes. Then, this effect may cause the development of a (111) texture component in accordance with the observation for CYS03 and CYS04.

The coverage of the crystal planes by sulfur atoms and cysteine molecules can influence not only the texture, but also the grain size and the defect density. If sulfur atoms are adsorbed on the surface of a growing crystal during deposition, the strong Ni–S covalent bonds and their preferred bonding angles [60] can increase the probability of grain boundary formation. Similarly, the cysteine molecules attached to the surface can form an adsorbed monolayer acting as an “organic shield” on the crystal planes, thereby hindering the ion transfer, but not blocking the electron transfer (though increasing the overvoltage of the Ni^2+^ ion reduction process at the same time). Thus, a decrease of the grain size was observed due to cysteine addition to the bath. Twin faults are special grain boundaries with low energy. Therefore, when a new crystal is nucleated on a surface for which the growth was blocked by sulfur atoms and/or cysteine molecules, the new and old crystals are often separated by coherent twin faults. In addition, the non-coherent grain boundaries may contain dislocations. Thus, both the dislocation density and the twin fault probability increased as a result of cysteine addition (see Figure 4).

It is worth comparing the microstructure developed in the present Ni layers deposited from cysteine containing bath with Ni films processed with other additives, such as saccharin, nickel-chloride or trisodium citrate [8]. Our former study revealed that the smallest grain size and the highest defect density were obtained when saccharin was added to the electrolyte bath [8]. For the present Ni samples processed with 0.3–0.4 g/L cysteine, similarly small grain size (20–27 nm) and twin fault probability (about 3%) were achieved; however the dislocation density was much higher (∼620 × 10^14^ m^-2^) than that for the former Ni deposited from the saccharin-containing bath (∼160 × 10^14^ m^-2^). In nanocrystalline materials, the majority of dislocations can be found in the grain boundaries [67]. Since the grain size is the same for both Ni films processed with saccharin and cysteine while the dislocation density is much higher for the CYS04 sample, a unit area in the grain boundaries contains more dislocations in the latter specimen. Such dislocations may form in order to relax the stresses caused by the segregation of additives to the grain boundaries. Therefore, the higher dislocation density in the Ni film processed with cysteine suggests that a higher distortion was developed at the grain boundaries during electrodeposition compared to the layer produced from the saccharin-containing bath.

### 4.2. Influence of Concentration of Cysteine on the Hardness

Figure 6 shows that the hardness increased monotonously with increasing cysteine content of the bath and a saturation was achieved at 0.2 g/L. The enhancement of the hardness due to the addition of 0.1–0.2 g/L cysteine can be attributed to the reduced grain size, the increased defect density and the development of (111) texture. Indeed, former studies have shown that (111) texture yielded a higher hardness than that for other crystallographic directions in fcc metals [68,69]. Although the defect density increased when the cysteine content was raised from 0.2 to 0.3 g/L, further enhancement in the hardness was not observed. Most probably, the defect-induced hardening was compensated by the texture softening, since for the cysteine contents of the 0.3–0.4 g/L texture component (002) became dominant (see Figure 5). A significant difference between the hardness values for 0.3 and 0.4 g/L cysteine contents was not observed, since all microstructural parameters and the texture for these two samples agree within the experimental error.

The hardness of the CYS04 sample (∼6800 MPa) is considerably higher than that for any other Ni film deposited formerly with additives [8]. In this previous study, it was found that saccharin addition caused the highest hardness among the Ni layers processed with different organic additives. The present investigation revealed that, for comparable grain sizes, cysteine may cause a hardness even higher than the saccharin additive. The difference between the grain boundary structures of the Ni films processed with cysteine and saccharin might have caused the higher hardness for the former sample. Indeed, the emission of dislocations from grain boundaries decorated with a high amount of additive atoms and/or molecules is difficult during hardness testing since dislocations help to relax the stresses caused by these additives, i.e., there is an attractive interaction between the dislocations and the additives in the boundaries. Therefore, the plastic deformation is difficult, resulting in an elevated hardness for the CYS04 sample.

### 4.3. Effect of Cysteine on the Thermal Stability

Figure 12 shows that the hardness of the Ni film processed with cysteine did not decrease up to 600 K or even increased significantly, by 24% at 500 K. The latter phenomenon has already been observed for Ni and Ni alloy electrodeposits and called anneal-hardening [24,70,71,72,73,74,75,76,77,78]. This effect was explained by the structural relaxation of grain boundaries (e.g., by annihilation of excess dislocations which are geometrically not necessary) and the segregation of impurities and solute atoms to grain boundaries in alloy samples. These changes in the grain boundaries result in a more difficult emission of dislocations from grain boundaries and a hindered grain boundary sliding, which are the main deformation mechanisms in nanocrystalline materials with larger and smaller grains, respectively. Due to the impeded plastic deformation, hardening occurs which depends on the grain size. The smaller the grain size, the higher the anneal-hardening effect. For Ni with a grain size of about 30 nm, the hardness increased by 15%; however, when the grain size decreased to about 3 nm (for Ni–Mo electrodeposits), the hardness enhancement reached 120% [70]. Moreover, the temperature of annealing, for which the maximum hardness was observed, increased from 500 to 800 K when the grain size decreased from 30 to 3 nm (the duration of heat treatment was 1 h for all samples) [70].

**Figure 13:**
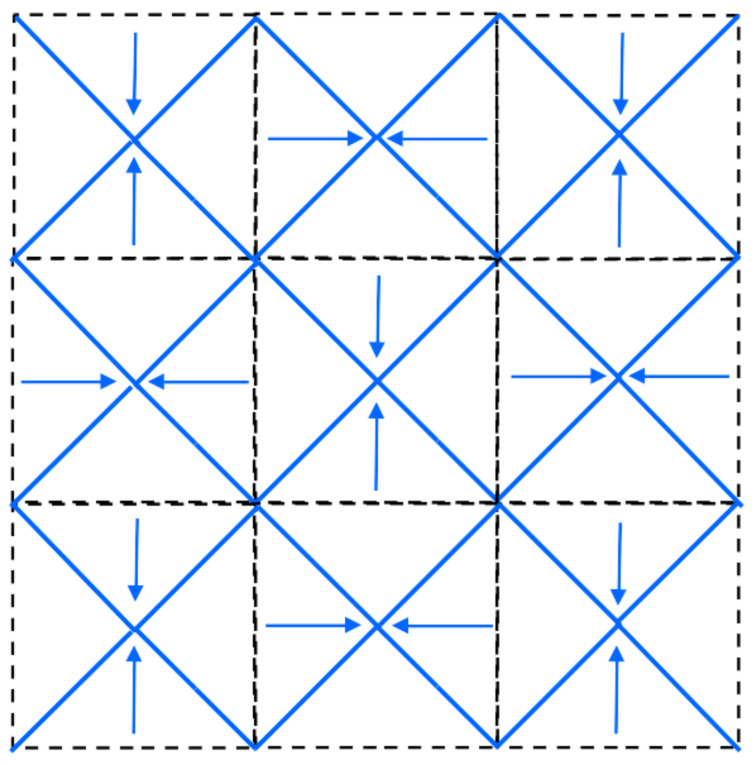
A schematic showing how the grain-boundary faceting can cause the reduction of the grain size. The black dashed lines illustrate the original grain boundaries while the blue solid lines represent the new grain boundaries after faceting. The arrows indicate the movement direction of the boundaries.

It should be noted that in the former studies, anneal-hardening is accompanied by a slight grain growth, but the hardening caused by the change of grain boundaries compensated the softening due to coarsening. At the same time, in the present case, heating of the sample CYS04 up to 500 K resulted in a decrease of the grain size from ∼26 to ∼15 nm (see Figure 9). This is an interesting observation, since the annealing of nanocrystalline materials usually results in grain coarsening. However, the following thermodynamic consideration may explain this unusual behaviour. Namely, the segregation of impurities and solute alloying elements (both of them can be called foreign atoms) can decrease the free energy of grain boundaries [79]. For a given foreign atom concentration and temperature, the minimum free energy of the material is achieved at a certain grain size (i.e., for a certain grain boundary area) [80]. If the grain size obtained during processing is larger than this optimal value, there is a thermodynamic driving force for the reduction of the grain size, i.e., for the increase of the amount of grain boundaries. It was found that the grain boundary area can increase by faceting of the existing boundaries without the nucleation of new grains [81]. The faceting of boundaries can be achieved by grain boundary migration, therefore this phenomenon may occur at relatively low temperatures of annealing. If the faceted grain boundaries come in contact with each other, grain size reduction may occur, as it was observed in the present case. Figure 13 shows a model of grain refinement caused by faceting of grain boundaries.

The grain refinement at 500 K may be expected to contribute to the hardening, since for nanocrystalline Ni, the Hall–Petch formula is valid down to the grain size of about 14 nm [82]. On the other hand, significant twin fault probability was found in this sample by XLPA and twin faults are similarly strong obstacles against dislocation glide as general grain boundaries. Therefore, when the twin fault spacing is smaller than the grain size, the former value is suggested to be used in the Hall–Petch formula. The twin fault spacing can be obtained from the twin fault probability as $100\times d_{111}/\beta$, where d111 is the lattice spacing for planes (111). Since for sample CYS04 annealed at 500 K, the twin fault spacing (∼9 nm) is lower than the grain size (∼15 nm), the decrease of the grain size has no contribution to the increase of the hardness. It is noted that the twin fault spacing slightly increased when film CYS04 was heated up to 500 K.

**Figure 14:**
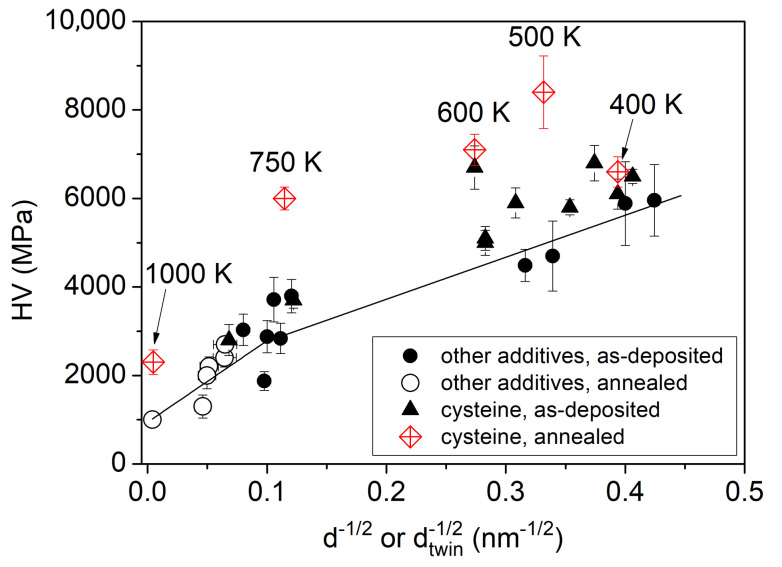
Hall–Petch plot for the films processed with cysteine for both the as-deposited and annealed states. In addition, the hardness values obtained on Ni films processed with other additives (e.g., saccharin and trisodium citrate) are also shown for comparison. from Ref. [9]. The two straight lines for grain sizes smaller and larger than 100 nm were obtained by fitting to the data determined for the Ni films processed with additives other than cysteine (published in [9]).

For revealing the correlation between the microstructure and the hardness, the Hall–Petch plot for the films processed with cysteine is shown in Figure 14. The data obtained on the heat-treated samples are also plotted. In addition, the hardness values obtained on Ni films processed with other additives (e.g., saccharin and trisodium citrate) are also shown. These data were taken from Ref. [9]. It can be seen that the data points obtained for as-deposited films from a cysteine-containing bath are lying only at a slightly higher hardness level than the earlier data reported for as-deposited Ni layers processed with the use of other additives. At the same time, the samples processed with cysteine and heat-treated at 500 K or higher temperatures exhibit considerably higher hardness than that predicted by the Hall–Petch plot obtained for Ni films, either as-deposited or annealed, prepared by using other additives. This observation suggests that the annealing of the specimens deposited from cysteine containing bath increased the strength of the grain boundaries, i.e., the deformation mechanisms (e.g., dislocation emission from the boundaries) became more difficult due to the change of the grain boundary structure, as discussed in the first paragraph of this section. The increase of the dominance of (111) texture during annealing of the samples processed with cysteine might have also contributed to the high hardness of the heat-treated films compared to the Hall–Petch trend determined for other specimens.

As demonstrated by Figure 12, the thermal stability of Ni films processed with cysteine is much better than that for other additives. Indeed, the hardness remained as high as ∼6000 MPa even at 750 K, while for other additives, the hardness fell below ∼3000 MPa at such high annealing temperatures. The higher hardness of the Ni film deposited from the cysteine-containing bath can be explained by the more stable grain size and defect density. Indeed, while for other additives the grain size increased above ∼240 nm at 750 K [9], for the present sample, the grain size remained below 100 nm. Similarly, the remaining dislocation density at 750 K was about one order of magnitude higher for CYS04 sample than for the other Ni films deposited with other additives.

## 5. Conclusions

Nanocrystalline Ni electrodeposits with cysteine additive were successfully processed. The microstructure and the hardness as a function of the cysteine content were investigated. In addition, the thermal stability of the Ni film produced with the highest applied cysteine content was studied. The following conclusions were drawn from the results:

1. The optimal parameters for electrodeposition of nickel from a cysteine-containing bath differ from the ones used for deposition from a conventional nickel-sulfate based bath. A nearly neutral pH with the value of 6.1, a high current density of –25 mA/cm^2^ and an increased concentration of boric acid (30 g/L) in the bath are recommended for obtaining nice deposits with cysteine.
2. With increasing the cysteine content up to 0.3 g/L, the density of lattice defects (dislocations and twin faults) increased, while the grain size decreased in the Ni films. In addition, the (220) texture in the additive-free Ni layer changed to (200) texture. When the cysteine content was enhanced from 0.3 to 0.4 g/L, further significant variation in the microstructure was not observed.
3. The Ni film obtained with the addition of 0.4 g/L cysteine exhibited a higher hardness (∼6800 MPa) than the values reported for other additives in the literature. The thermal stability of the nanostructured Ni film fabricated with cysteine was exceptional; namely, the hardness of the Ni layer deposited from cysteine-containing bath remained as high as ∼6000 MPa, even after heating up to 750 K. The relatively large hardness was retained upon annealing even at 1000 K, while the (111) texture became stronger.
4. When the Ni film processed with cysteine was heated up to 500 K, the grain size decreased from ∼27 to ∼15 nm and the hardness increased to ∼8400 MPa, which is an exceptionally high value among Ni electrodeposits. This very high hardness might be attributed rather to the structural changes in the grain boundaries and the strengthening of the (111) texture than to the reduction of the grain size.

## Figures and Tables

**Table 1:** Composition of the electrolyte bath containing cysteine.

Component	Concentration (g/L)	Role
NiSO_4_·6H_2_O	155	Ni source
H_3_BO_3_	30	pH-buffer
NaSO_4_·10H_2_O	96	Supporting electrolyte
H_2_NSO_3_H	10	wetting agent
Cysteine HSCH_2_CH(NH_2_)COOHS	0; 0.1; 0.2; 0.3; 0.4	additive
pH Current density	6.1 −25 mA/cm^2^	

**Table 2:** Microstructural parameters and Vickers hardness (HV) of samples deposited from baths containing different amounts of cysteine. Both the electrolyte side (es) and the substrate side (ss) were investigated. The table contains the crystallite size (<*x*>), the dislocation density (ρ) and the twin fault probability (β) obtained by XLPA, as well as the mean grain size obtained from TEM (*d*_*TEM*_).

Concentration (g/L)	Side	<*x*> (nm)	ρ ($10^{14}m^{-2}$)	β (%)	*d*_TEM_ (nm)	HV (MPa)
0	es	64 ± 6	34 ± 4	0 ± 0.1	215 ± 70	2800 ± 350
0	ss	23 ± 3	39 ± 5	0 ± 0.1	67 ± 12	3700 ± 180
0.1	es	34 ± 3	380 ± 40	1.6 ± 0.2	37 ± 3	5100 ± 270
0.1	ss	36 ± 4	360 ± 40	1.6 ± 0.2	38 ± 2	5000 ± 280
0.2	es	25 ± 3	300 ± 30	1.5 ± 0.2	19 ± 4	6700 ± 490
0.2	ss	28 ± 3	360 ± 40	1.9 ± 0.2	31 ± 3	5900 ± 340
0.3	es	28 ± 3	550 ± 60	2.5 ± 0.3	21 ± 3	5800 ± 170
0.3	ss	28 ± 3	550 ± 60	3.1 ± 0.3	20 ± 3	6100 ± 340
0.4	es	27 ± 3	620 ± 70	2.8 ± 0.3	27 ± 3	6800 ± 400
0.4	ss	28 ± 3	610 ± 60	3.3 ± 0.3	25 ± 3	6500 ± 160

**Table 3:** The difference between the conditions of electrodeposition of the additive-free sample from Ref. [8] (denoted as “NOA”) and the additive-free sample used in this study (denoted as “CYS00”) and a comparison between the microstructural parameters, texture and hardness.

Sample	Boric Acid (g/L)	pH	Curr. Dens. (mA/cm^2^)	<*x*> (nm)	ρ ($10^{14}m^{-2}$)	β (%)	*d*_TEM_ (nm)	HV (MPa)	Texture
NOA-es	15	3.25	−6.25	42 ± 5	12 ± 1	0 ± 0.1	127 ± 45	1877 ± 211	(220)
NOA-ss	15	3.25	−6.25	27 ± 3	14 ± 2	0 ± 0.1	91 ± 14	2837 ± 344	NA
CYS00-es	30	6.1	−25	64 ± 6	34 ± 4	0 ± 0.1	215 ± 70	2800 ± 350	(220)
CYS00-ss	30	6.1	−25	23 ± 3	39 ± 5	0 ± 0.1	67 ± 12	3700 ± 180	NA

**Table 4:** The microstructural parameters and the hardness of the samples deposited from the bath containing 0.4 g/L cysteine and heat treated up to different temperatures. Only the electrolyte side (es) of the samples was investigated. The table contains the mean crystallite size (<*x*>), the dislocation density (ρ) and the twin fault probability (β) obtained XLPA, the mean grain size obtained by TEM (*d*_TEM_) and the Vickers hardness (HV).

Temperature (K)	<*x*> (nm)	ρ ($10^{14}m^{-2}$)	β (%)	*d*_TEM_ (nm)	HV (MPa)
300	27 ± 3	620 ± 70	2.8 ± 0.3	27 ± 3	6800 ± 400
400	28 ± 3	720 ± 70	3.1 ± 0.3	26 ± 2	6600 ± 340
500	11 ± 2	570 ± 60	2.2 ± 0.2	15 ± 2	8400 ± 820
600	58 ± 6	340 ± 40	1.5 ± 0.2	24 ± 2	7100 ± 350
750	88 ± 14	91 ± 10	0 ± 0.1	76 ± 6	6000 ± 260
1000	NA	NA	NA	43,000 ±6600 ^1^	2300 ± 280

^1^ Grain size for sample annealed up to 1000 K was determined by EBSD.
